# Efficient Gene Silencing by Self-Assembled Complexes of siRNA and Symmetrical Fatty Acid Amides of Spermine

**DOI:** 10.3390/pharmaceutics3020125

**Published:** 2011-03-25

**Authors:** Abdelkader A. Metwally, Charareh Pourzand, Ian S. Blagbrough

**Affiliations:** Department of Pharmacy and Pharmacology, University of Bath, Bath BA2 7AY, UK

**Keywords:** fatty acids, gene silencing, GFP, lipoplexes, self-assembly, siRNA, spermine

## Abstract

Gene silencing by siRNA (synthetic dsRNA of 21-25 nucleotides) is a well established biological tool in gene expression studies and has a promising therapeutic potential for difficult-to-treat diseases. Five fatty acids of various chain length and oxidation state (C12:0, C18:0, C18:1, C18:2, C22:1) were conjugated to the naturally occurring polyamine, spermine, and evaluated for siRNA delivery and gene knock-down. siRNA delivery could not be related directly to gene silencing efficiency as *N*^4^,*N*^9^-dierucoyl spermine resulted in higher siRNA delivery compared to *N*^4^,*N*^9^-dioleoyl spermine. GFP silencing in HeLa cells showed that the unsaturated fatty acid amides are more efficient than saturated fatty acid amides, with *N*^4^,*N*^9^-dioleoyl spermine resulting in the most efficient gene silencing in the presence of serum. The alamarBlue cell viability assay showed that fatty acid amides of spermine have good viability (75%–85% compared to control) except *N*^4^,*N*^9^-dilauroyl spermine which resulted in low cell viability. These results prove that unsaturated fatty acid amides of spermine are efficient, non-toxic, non-viral vectors for siRNA mediated gene silencing.

## 1. Introduction

siRNA is a synthetic double-stranded (dsRNA) of 21-25 nucleotides per strand. Post-transcriptional gene silencing by siRNA is an important biological tool in functional genomics studies and has many potential therapeutic applications for difficult-to-treat diseases. After intracellular entry and subsequent release of siRNA in the cytoplasm, siRNA mediates sequence specific mRNA degradation. The process of gene silencing starts when siRNA is loaded in the RNA induced silencing complex (RISC), a complex of siRNA and other proteins (enzymes) among which is the endonuclease argonaute-2, the enzyme that catalyzes the degradation of mRNAs that have a complementary sequence to the siRNA loaded in the RISC. During the RISC loading, the passenger siRNA strand is cleaved and only the guide (anti-sense) strand is loaded. mRNA possessing a complementary sequence to the guide strand is then cleaved by the RISC. siRNA is a potential therapeutic for the treatment of many diseases such as thyroid papillary carcinoma [1] and osteoclast-mediated bone resorption [2], recently reviewed by Blagbrough and Zara [3]. Many clinical trials that involve siRNA or vectors expressing shRNA are now in phase I and phase II, e.g. RNAi assessment of Cand5 in diabetic macular edema (RACE) (Phase II), using intra vitreal injections of a siRNA in patients with age-related macular degeneration (Phase II), and a phase I open-label, rising-dose study of the safety and tolerability of single doses of NUC B1000 (http://www.wiley.com/legacy/wileychi/genmed/clinical/) which is an RNAi-based therapy for chronic hepatitis B.

Nanoparticle formulation of siRNA as a platform for therapeutic applications has very recently been reviewed in this journal [4]. Cationic lipids are among the most widely used non-viral gene delivery vectors [5-12]. Cationic lipids are amphipathic molecules that are composed of a hydrophobic part and a cationic head group which may be attached to the hydrophobic part directly or through a linker [13,14]. The hydrophobic part of cationic lipids varies from alkyl or fatty acid (acyl) chains [15-17] to steroids, e.g. cholesterol [18,19]. The cationic head group should contain one or more functional groups that can acquire a positive charge at a physiological pH of 7.4 such as amines (primary, secondary, tertiary, and quaternary) [15,20], guanidines, imidazoles, or pyridinium salts [20]. The positive charge on the cationic head group interacts electrostatically with the negative charge of the phosphate backbone of the siRNA while the hydrophobic part of the cationic lipids promotes the formation of vesicles or siRNA/cationic lipid aggregates called lipoplexes. The hydrophobic moiety of cationic lipids can affect the siRNA delivery and the biological activity (gene knock-down) according to its physical and chemical properties such as chain length and saturation state.

We have therefore designed a series of symmetrical diacyl lipopolyamines in order to prepare lipoplex formulations (without any pre-preparation of liposomes) of an Alexa Fluor 647-tagged siRNA to investigate if they are suitable for non-toxic transfection of target cells, by forming nanoparticles which will efficiently enter cells for non-viral gene therapy (NVGT) either by endocytosis (a major pathway) or possibly by endocytosis in combination with fusion between siRNA lipoplexes and the plasma membrane (a minor pathway) [9]. Such formulations of lipoplexes lead to RNA knock-down in which the siRNA binding is achieved by anion titration. Herein we report our investigations on formulations of siRNA with variation in the length of the two acyl fatty chains regiospecifically covalently bound to spermine. We report the synthesis of five symmetrical diacyl lipopolyamines, varying the chain length from lauroyl C12 to the very long chain erucoyl C22, and varying the oxidation (saturation) state by including stearoyl (18:0), oleoyl (18:1), and linoleoyl (18:2) as well as the unsaturated erucoyl (22:1) spermine conjugate. We provide detailed evidence for the characterization of the nanoparticles, the delivery of Alexa Fluor 647-tagged siRNA and the silencing of GFP biosynthesis, the cell viability, and we compare our results with those obtained with cationic liposomal Lipofectamine 2000 and non-liposomal TransIT TKO.

## 2. Experimental Section

### 2.1. Materials and general methods

Dicyclohexylcarbodiimide (DCC), 4-dimethylaminopyridine (DMAP), fatty acids, fatty acid chlorides, G418, hydrazine monohydrate, *N*-carbethoxyphthalimide, spermine, and triethylamine (TEA), were purchased from Sigma-Aldrich (Gillingham, UK). All solvents were purchased from Fisher Scientific UK (Loughborough, UK). Cell culture media were purchased from Gibco (Invitrogen Ltd, Paisley, UK). HeLa cells stably expressing GFP were obtained from the Cell Service at Cancer Research UK (CRUK, London Research Institute, Clare Hall Laboratories, South Mimms, London, UK). NMR spectra were recorded in chloroform-D using a Varian Mercury 400 (operating at 400 MHz for ^1^H and 100.8 MHz for ^13^C) spectrometer. The high resolution (HR) time-of-flight mass spectra were obtained on a Bruker Daltonics micrOTOF mass spectrometer using electrospray ionisation (ESI). AllStars negative control siRNA tagged with Alexa Fluor^®^ 647 at the 3′-position was purchased from Qiagen (Crawley, UK) as was siRNA against GFP labelled with Alexa Fluor^®^ 647 at the 3′-position of the sense strand, sequences:

Sense strand: 5′-GCAAGCUGACCCUGAAGUUCAUTT-3′,

Anti-sense strand: 5′-AUGAACUUCAGGGUCAGCUUGCCG-3′,

Target DNA sequence: 5′-CGGCAAGCTGACCCTGAAGTTCAT-3′.

### 2.2. Synthesis of fatty acid amides of spermine

*N*-Carbethoxyphthalimide (0.44 g, 2 mmol) was added to a solution of 1,12-diamino-4,9-diazododecane (spermine) (0.20 g, 1 mmol) in DCM (10 mL). The solution was stirred 20 °C for 3 h then evaporated to dryness in vacuo and the residue was used directly in the following step. To a solution of 1,12-diphthalimido-4,9-diazadodecane in DCM (10 mL) and TEA (0.28 mL, 2 mmol) fatty acid chloride (2 mmol), or alternatively fatty acid (2 mmol), DMAP (0.24 g, 2 mmol), and DCC (0.4 g, 2 mmol) were added and stirred for 18 h under nitrogen atmosphere. The solvent was then evaporated to dryness *in vacuo* and the residue was treated with hydrazine monohydrate (2 mL) in a mixture of DCM (15 mL) and THF (15 mL) and heated under reflux for 4 h then the solvent was evaporated in vacuo to dryness and the residue purified over silica gel (DCM/MeOH 10:1 v/v then DCM/MeOH/ NH_4_OH 20:10:1 v/v/v) to afford the title compounds, which were then fully characterized by both high-field ^1^H and ^13^C NMR spectroscopy and HR-ESI-MS. ^1^H and ^13^C NMR data were comparable with literature data [21-23]. 1,12-Diphthalimido-4,9-diazadodecane, HRMS m/z, ESI found (M+1)^+^ 463.2354, C_26_H_31_N_4_O_4_ requires (M+1)^+^ 463.2340. *N*^4^,*N*^9^-Dierucoylspermine, **1** [21], HRMS m/z, ESI found (M+1)^+^ 843.8355, C_54_H_106_N_4_O_2_ requires (M+1)^+^ 843.8316. *N*^4^,*N*^9^-Dilauroylspermine, **2** [22], HRMS m/z, ESI found (M+1)^+^ 567.5553, C_34_H_70_N_4_O_2_ requires (M+1)^+^ 567.5572. *N*^4^,*N*^9^-Dilinoleoylspermine, **3** [23], HRMS m/z, ESI found (M+1)^+^ 727.6851, C_46_H_86_N_4_O_2_ requires (M+1)^+^ 727.6824. *N*^4^,*N* ^9^-Dioleoylspermine, **4** [23], HRMS m/z, ESI found (M+1)^+^ 731.7174, C_46_H_90_N_4_O_2_ requires (M+1)^+^ 731.7137. *N*^4^,*N*^9^-Distearoylspermine, **5** [23], HRMS m/z, ESI found (M+1)^+^ 735.7428, C_46_H_94_N_4_O_2_ requires (M+1)^+^ 735.7450.

### 2.3. Transfection studies of HeLa cells stably expressing GFP

Cells were trypsinized at confluency 80–90% and were seeded at a density of 65,000 cells/well in 24-well plates and were incubated for 24 h at 37 °C, 5% CO_2_, prior to transfection. The lipoplexes were prepared by mixing the specified amounts of the transfection reagent in OptiMEM serum-free medium (50μL) with 15 μL of siRNA (1 μM) in OptiMEM serum-free medium. The solutions were mixed for 4 s by means of a vortex mixer. On the day of transfection, the lipoplex solutions were added to wells containing DMEM (10% FCS) to make the final volume in each well 1 mL. The plates were then incubated for 48 h at 37 °C, 5% CO_2_. siRNA against GFP used in these experiments has 24 base-pairs, thus each molecule of siRNA contains 48 negative charges corresponding to 48 negatively charged phosphate groups in the siRNA backbone. The synthesized spermine fatty acid amides each contain two terminal primary amine groups which will be positively charged at physiological pH 7.4, therefore, each vector molecule carries two positive charges. N/P ratio is calculated using the following equation:

$$N/P=\frac{\text{number of moles of cationiclipid}\times 2}{\text{number of molesof siRNA}\times 48}$$

### 2.4. Flow cytometry (FACS)

For analysis of delivery and then reduction of expression of GFP by flow cytometry, cells were trypsinized and resuspended in complete medium without phenol red. Cells were centrifuged (1,000 rpm for 5 min), and washed twice by resuspending in PBS containing 0.1% BSA (1 mg/mL bovine serum albumin) then and centrifugation (1,000 rpm for 5 min). The collected cells was then resuspended in PBS and transferred to a flow cytometer tube (Becton Dickinson, UK). Cells (typically 10,000–20,000 events) were then analyzed using a FACSCanto flow cytometer (Becton Dickinson, UK), equipped with an argon ion laser at 488 nm for excitation, a Long Pass (LP) filter at 502 nm and a detector at 530 nm (range +/−15 nm) for fluorescence emission, helium/neon laser at 633nm, and detector for the Alexa Fluor 647 at 660 nm (range +/− 10 nm). GFP expression is calculated as:

$$\%\text{GFP}=\frac{\text{GFP fluorescenceof transfected cells}}{\text{GFP fluorescence of control cells}}\times 100$$

### 2.5. Confocal microscopy cell imaging

Cells were trypsinized at confluency 80–90% and were seeded at a density of 65,000 cells/well in 24-well plates that have a round-glass cover slip (12 mm in diameter) and were incubated for 24 h prior to transfection which was carried out as described above (Section 2.3). After 48 h, the cell culture media, in each well, were aspirated and the cells washed with PBS (3 × 0.5 mL). The cell membrane was then stained with wheat germ agglutinin (WGA) conjugated to Alexa Fluor^®^ 555. The concentration of WGA-Alexa Fluor^®^ 555 working solution was adjusted to a concentration of 5 μg/mL in Hank's balanced salt solution without phenol red. The cells were incubated for 10 min in the dye working solution at 37 °C, 5% CO_2_ in the dark. The cells were washed with PBS (3 × 0.5 mL) and then fixed with 4% paraformaldehyde in PBS solution for 20 min at 20 °C in the dark. The cover slips were then removed from each well, washed with PBS (2 × 0.5 mL), left to dry briefly in air, and then mounted on glass slides using Mowiol (polyvinyl alcohol) solution as the mounting media and left in the dark at 20 °C (18 h) to allow hardening of the mounting media. The cells were examined using a Carl Zeiss laser scanning microscope LSM 510 meta, with GFP excitation 488 nm, emission 505–550 nm (band pass filter), Alexa Fluor^®^ 555 excitation 543 nm, emission 560–615 nm (band pass filter), and Alexa Fluor^®^ 647 excitation 633 nm, emission 657–753 nm (meta detector).

### 2.6. Cell viability assay

Cells were seeded at a density of 6,500 cells per well of 96-well plates. The transfection was carried out using the same protocol as transfecting the 24-well plates with the exception of reducing the amount of lipoplexes such that each well contains 1.5 pmol siRNA in a final volume of 100 μL/well. After 44 h, alamarBlue® [24] (10 μL) was added to each well. After incubation (3.5 h), the absorbance of each well was measured at 570 nm and 600 nm and calculations were carried out according to the standard protocol provided by the supplier.

### 2.7. Particle size and zeta potential measurements

Lipoplexes were prepared by adding siRNA solution (75 μL, 1 μM) in HEPES (pH 7.4, 10 mM) to HEPES (250 μL) containing the specified amount of transfection reagent followed by vortex mixing for 4 s. Samples were then diluted to a final volume of 3 mL by HEPES buffer. Samples were mixed for 10 s directly before measurements. Measurements were carried out using Malvern Zetasizer Nano S90 using refractive index 1.59, viscosity 0.89 cP, dielectric constant 79, and temperature set to 25 °C with equilibrium time 3 min. Z-Average diameter in nm and zeta potential in mV were recorded as averages of three and six measurements respectively.

## 3. Results and Discussion

### 3.1. Synthesis of fatty acid amides of spermine

**Figure 1.**
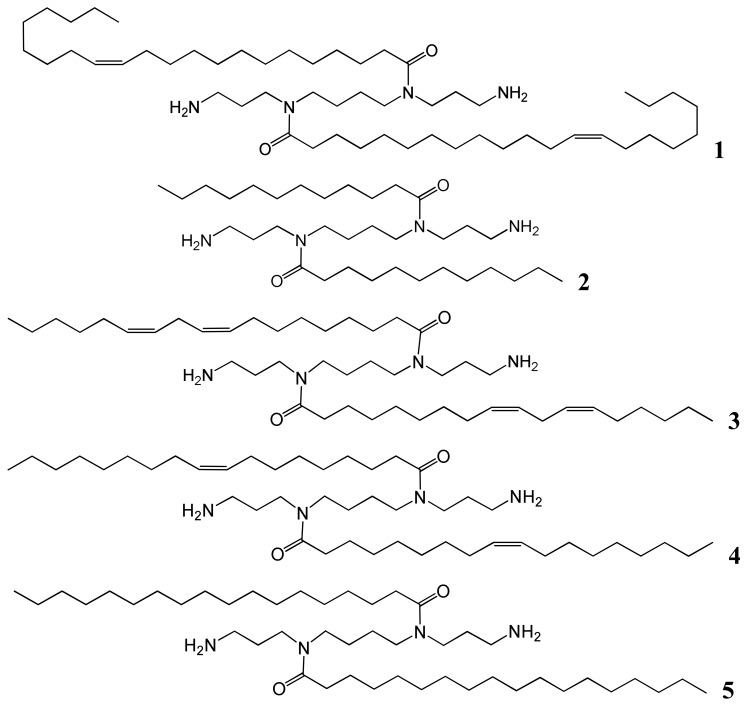
*N*^4^,*N*^9^-Difatty acid amides of spermine.

Spermine (*N*,*N*′-bis(3-aminopropyl)-1,4-diaminobutane) was used as the starting material to synthesize the five desired fatty acid amide conjugates: *N*^4^,*N*^9^-dierucoyl spermine **1**, *N*^4^,*N*^9^-dilauroyl spermine **2**, *N*^4^,*N*^9^-dilinoleoyl spermine **3**, *N*^4^,*N*^9^-dioleoylspermine **4**, and *N*^4^,*N*^9^-distearoyl spermine **5** (Table 1 and Figure 1). The two terminal primary amine groups of spermine were chemo-selectively protected with phthalimido protecting groups (2 eq. in CH_2_Cl_2_). The fatty acids were conjugated to the *N*^4^- and *N*^9^-position of the diphthalimido protected spermine by one of two methods, either fatty acyl chloride (lauroyl, oleoyl, or stearoyl) (2 eq.) and triethylamine (2 eq.) or alternatively fatty acid (erucic or linoleic) (2 eq.), DCC (2 eq.), DMAP (2 eq.) were added to the diphthalimido protected spermine solution in anhydrous CH_2_Cl_2_. After the completion of conjugation reaction as followed by TLC, the solution is filtered and residue is dissolved in 1:1 mixture of CH_2_Cl_2_/THF. Refluxing with hydrazine (2 mL) resulted in deprotection of the primary amine groups. The required fatty acid conjugates of spermine were purified by flash silica column chromatography to homogeneity by TLC. Confirmation of the structures of the pure target cationic lipids **1-5** followed from HRMS and comparison of their ^1^H and ^13^C NMR spectroscopic data with those in the literature [21-23].

### 3.2. Lipoplex particle size and ζ-potential

The particle size and **ζ**-potential (Table 1) measurements were carried out on the lipoplexes at the cationic lipid/siRNA ratio which gave the best knock-down for the specified fatty acid amide of spermine. Particle size characterization by dynamic light scattering showed that the particle size of the formed lipoplexes varied from 145 to 353 nm which is within the size range for lipoplexes required for siRNA and/or DNA delivery, probably via endocytosis. The largest lipoplex diameter (353 nm) was obtained with **3** while the smallest (145 nm) was obtained with **5**. There was no obvious direct relationship between the lipoplex particle size and either the ratio of cationic lipid/siRNA or the characteristics (chain length and/or oxidation state) of the fatty acid used. The ζ-potentials of the lipoplexes were all positive (+25–63 mV), thus playing a role in the stabilization of the lipoplexes by imparting repulsion between the formed lipoplexes and preventing their aggregation. The positive charge of the lipoplexes also facilitates interaction with the negatively charged cell membrane.

### 3.3. Transfection with siRNA and evaluation of delivery and knock-down

**Figure 2.**
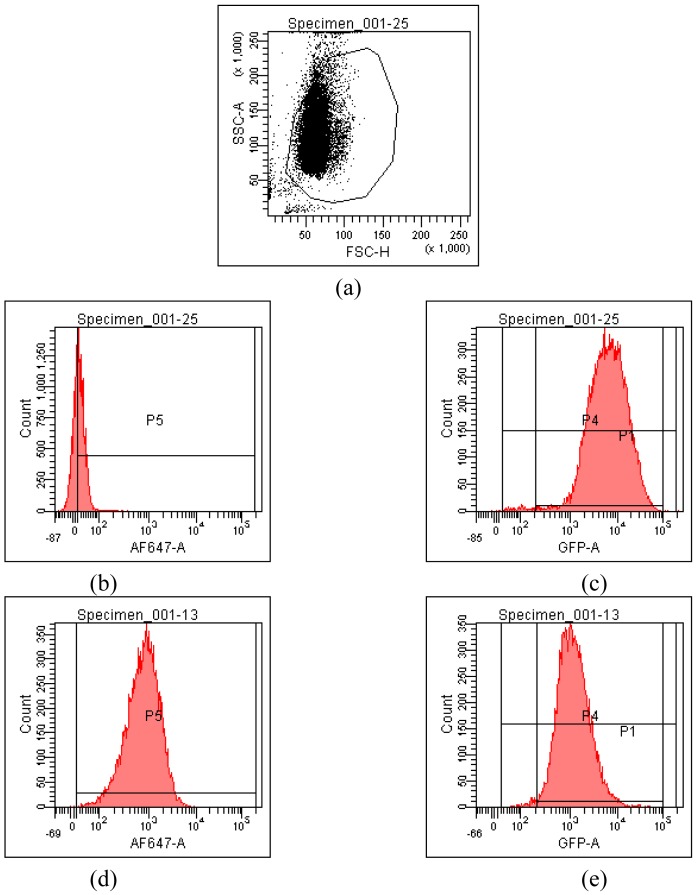
(**a**) The gating of a population of healthy cells. (**b**) Alexa Fluor 647 channel autofluorescence of control cells. (**c**) GFP expression of control cells. (**d**) Alexa Fluor 647 fluorescence after transfection with **4** at 6 μg/well. (**e**) GFP expression after transfection with **4** at 6 μg/well.

**Figure 3.**
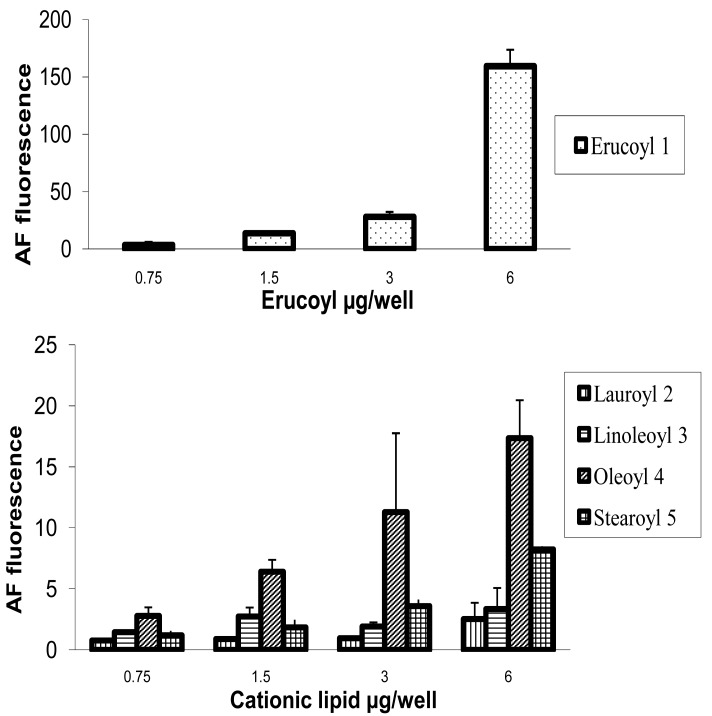
**Upper**: siRNA delivery using **1**. Values are presented as mean of normalized geometric mean fluorescence ±SD (n = 6). **Lower**: siRNA delivery measured as normalized values of Alexa Fluor 647 (AF) geometric mean fluorescence using **2-5**.

HeLa cells [25] are often used as a target for proof of principle of transfection efficiency [8,26]. Lipoplexes prepared with the synthetic fatty acid spermine conjugates **1**-**5** and siRNA tagged with the fluorescent dye Alexa Fluor 647 were delivered to modified HeLa cells that stably express GFP in order to evaluate both the delivery of siRNA and the knock-down of GFP expression. Both the delivery of siRNA and GFP knock-down were evaluated by calculating the geometric mean fluorescence of Alexa Fluor 647 and GFP by means of flow cytometry (FACS analysis) after gating the population of healthy cells (Figure 2a). The increase in Alexa Fluor 647 fluorescence within the gated population (*i.e.* delivery of fluorescent siRNA) and the decrease in GFP fluorescence (*i.e.* knock-down) are shown in Figure 2b-2e. Populations P4 and P5 are set for the calculation of the geometric mean fluorescence. The amount of Alexa Fluor 647 fluorescence 48 h post-transfection was used to evaluate the relative efficiency of siRNA delivery by the lipospermine conjugates. Lipoplexes of **1** showed the highest Alexa Fluor 647 fluorescence (159 units) at 6 μg of **1** (Figure 3, upper). Lipoplexes of **4** were second (17 units) at 6 μg of **4** (Figure 3, lower). Typically, there is a relationship between the amount of each fatty acid spermine conjugate and the intensity of the Alexa Fluor 647 fluorescence measured, except for **2** at 0.75–3 μg and for **3** at 1.5 and 3 μg. Thus, increasing the amount of cationic lipid, from 0.75 to 6 μg, gave an increase in Alexa Fluor 647 fluorescence (Figure 3, upper and lower).

Fatty acids with longer chain lengths and one C=C (erucoyl 22:1 and oleoyl 18:1) resulted generally in higher fluorescence at the same amount of lipid used. The least fluorescence was obtained with **2** (lauroyl 12:0) (Figure 3, lower) which is saturated and has a relatively shorter chain length.

**Figure 4.**
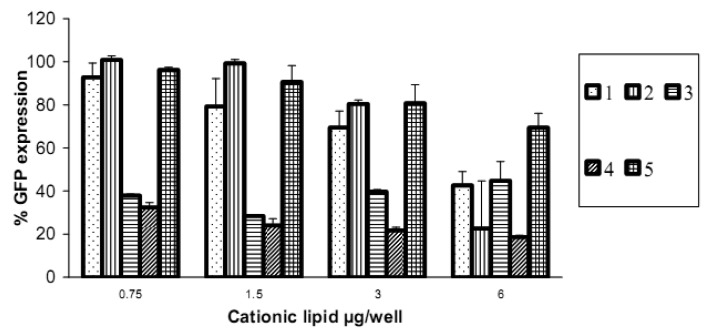
Reduction in GFP expression in HeLa cells after transfection with lipoplexes of fatty acid amides of spermine at different cationic lipid/siRNA ratios. siRNA concentration is kept constant at 15 pmol/well. Values are presented as mean ±SD (n = 6).

**Figure 5.**
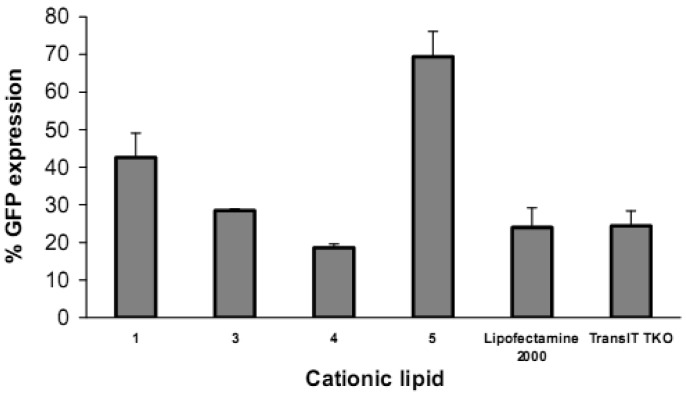
Comparison of reduction in GFP expression in HeLa cells (knock-down) at their optimal cationic lipid/siRNA ratios (shown as mg cationic lipid/15 pmol siRNA/well) and compared with the commercially available transfection agents Lipofectamine 2000 (2 μL/well) and TransIT TKO (4 μL/well). Values are presented as mean ±SD (n = 6) using erucoyl (6 μg/well), linoleoyl (1.5 μg/well), oleoyl (6 μg/well), and stearoyl (6 μg/well).

Reduction in GFP expression (Figure 4 and Figure 5) was best achieved by lipoplexes of **4** (19% at 6 μg cationic lipid and 22% at 3 μg) followed by **3** (28% at 1.5 μg). These values are comparable with the commercial agents Lipofectamine 2000 and TransIT TKO (both 24%). Lipoplexes of **1** resulted in modest reduction of GFP (43% at 6 μg). Lipoplexes of the saturated fatty acid conjugates **2** and **5** resulted in the lowest knock-down (highest GFP expression) with values of 80% and 69% respectively. The overall better efficiency of unsaturated fatty acid spermine amides in reducing GFP expression is attributed to the effects of double bonds restricting chain flexibility, allowing better interaction with the cell membrane lipids. We have reported [23] the effect of unsaturation of spermine fatty acid conjugates on their ability to transfect primary skin cell lines (FEK4, FCP4, FCP5, FCP7, and FCP8) and the cancer cell line HtTA transfected with pEGFP using the C18 derivatives of spermine linoleoyl (18:2), oleoyl (18:1), and stearoyl (18:0). The unsaturated oleoyl **4** and linoleoyl **3** spermine conjugates were found to be much more efficient (3-5-fold) than the stearoyl **5** spermine conjugate. Higher fusogenic ability of unsaturated fatty acids (especially in the *cis*-configuration) probably favors (L_α_ to H_II_) transition. A similar conclusion was reported [27] where unsaturated fluorinated lipospermines were more efficient than their saturated analogues due to the ability of unsaturated derivatives to promote membrane fusion and endosomal escape.

There was no obvious direct relationship between the particle size and the efficiency of delivery or knock-down. The particle size of lipoplexes formed with vectors **2** and **4** were 283 and 247 nm respectively, but **2** resulted in less delivery (3 units compared to 17 units of **4**) and lower reduction in GFP expression. Lipoplexes of **3** had larger particle size than **4** (353 and 247 nm respectively) and **3** showed less GFP reduction compared to **4**. On the other hand, though lipoplexes of **5** had the lowest diameter (145 nm), they showed less reduction in GFP (to 69%) compared to **3** and **4** (to 28% and to 19% respectively). These results show that, while keeping particle size within the normal lipoplex transfection range, particle size is not the only significant factor. Vectors **3**, **4**, and **5** have the same chain length while they differ in oxidation (saturation) state (18:2, 18:1, and 18:0 respectively).

The amount of siRNA delivered was evaluated by the fluorescence of Alexa Fluor 647 remaining after 48 h post transfection. There was no direct relationship between the amount of siRNA delivered and the knock-down efficiency. Lipoplexes of **1** resulted in the highest delivery (159 units at 6 μg) and GFP reduction (43%) while lipoplexes of **4** resulted in less siRNA delivery (17 units at 6 μg) and GFP reduction (19%) at the same amount of cationic lipid. The same can be said with respect to **3** (GFP reduction to 28% at 1.5 μg and delivery 3 units only) compared to **1**. However, with respect to vectors **1**, **4**, and **5**, the increase of Alexa Fluor 647 fluorescence is accompanied by better reduction in GFP. These observations can be related to the different barriers to successful knock-down. Lipoplexes must first be internalized inside the cells (probably by endocytosis) followed by release from the endosomes before turning into lysosomes, then the release of siRNA in the cytoplasm to integrate with the RNAi machinery and promote the sequence specific gene silencing. Thus, it is not a necessity that a higher number of lipoplexes (*i.e.* more siRNA gets into the cells) corresponds to an increase in gene knock-down, because successful knock-down will require the success of all the aforementioned steps.

**Figure 6.**
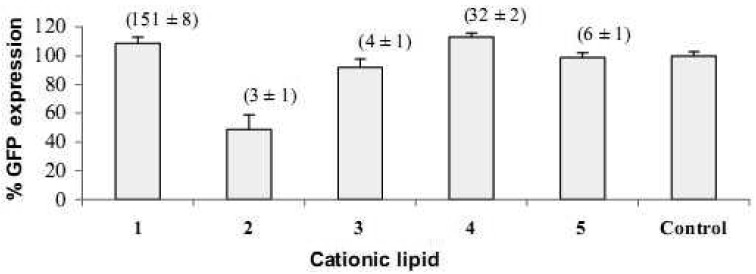
GFP percentage expression 48 h post transfection with scrambled negative control siRNA. Numbers in brackets are siRNA delivery expressed as Alexa Fluor^®^ 647 normalized geometric mean fluorescence ±SD.

Figure 6 shows the effect of transfecting HeLa cells with AllStars negative control siRNA at the optimum cationic lipid/siRNA ratios that resulted in the best GFP knock-down with respect to each of the spermine conjugate vectors. Although there was an increase in the levels of fluorescence of Alexa Fluor^®^ 647, there was no corresponding significant decrease in the GFP expression levels, except for compound **2** which is relatively toxic (as discussed below in Section 3.5). This indicates that the reduction of GFP fluorescence levels when transfecting with siRNA directed against GFP is due to the sequence-specific gene silencing mediated by the siRNA and not due to any cytotoxic or non-specified effects of the vector. The scrambled siRNA is reported by the manufacturer to lack homology to any mammalian genes and it also did not result in significant changes in GFP expression due to off-target effects. siRNA against GFP with sequence 5′-GCAAGCUGACCCUGAAGUUCAUTT-3′ sense strand and 5′-AUGAACUUCAGGGUCAGCUUGCCG-3′ anti-sense strand has one report of off-target effects that might affect gene-expression in HeLa and HEK cell lines [26]. The target DNA homology for silencing GFP is 5′-CGGCAAGCTGACCCTGAAGTTCAT-3′.

### 3.4. Confocal microscopy cell imaging

**Figure 7.**
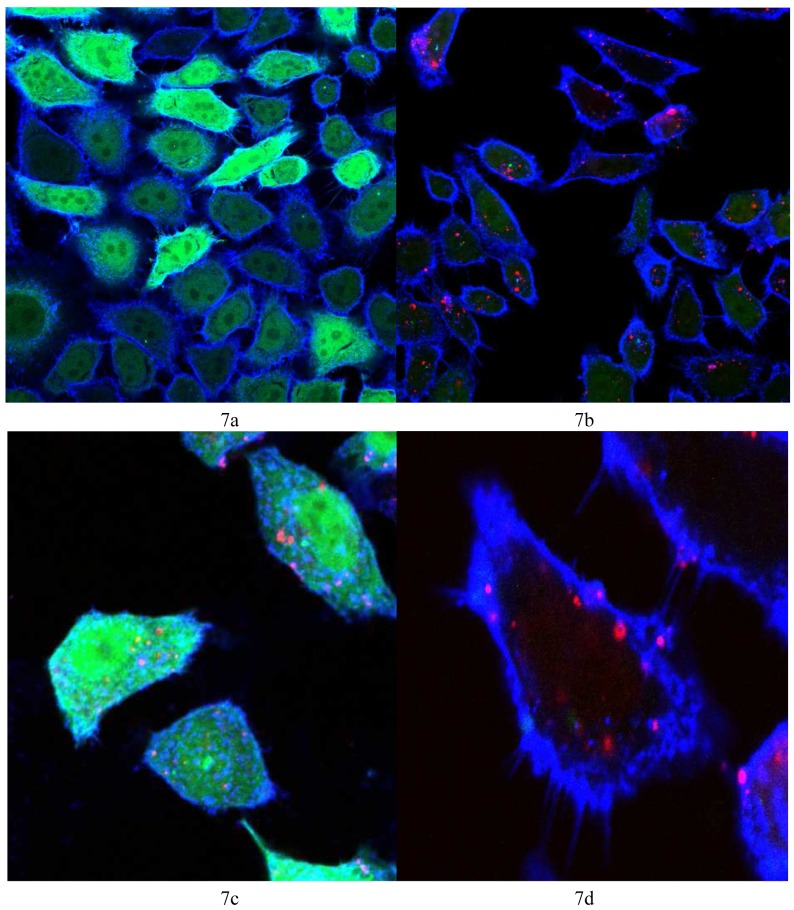
Confocal microscopy cell imaging. GFP fluorescence (green), cell membrane stained with WGA-Alexa Fluor^®^ 555 (blue), and Alexa Fluor^®^ 647 (red) represents tagged siRNA delivered with *N*^4^,*N*^9^-dioleoylspermine **4**. (**a**) non-transfected HeLa cells (control); (**b, d**) HeLa cells 48 h post transfection with siRNA against GFP; (**c**) HeLa cells transfected with AllStars negative control siRNA.

Vector **4** was used to prepare lipoplexes with Alexa Fluor^®^ 647 labeled AllStars negative control siRNA and siRNA against GFP at N/P = 22, the optimum ratio for the best GFP knock-down (6 μg/15 pmol siRNA). Figure 7a shows the control HeLa cells (non-transfected) showing the bright green fluorescence of GFP and the cell membrane stained with WGA-Alexa Fluor^®^ 555 (in blue). Figure 7b shows the HeLa cells 48 h post transfection where the fluorescence of GFP is significantly reduced and bright red spots indicate the fluorescence of Alexa Fluor^®^ 647 tagged siRNA, showing the uptake of the tagged siRNA. Figure 7d shows a magnified image of one cell (from Figure 7b) where many red spots show the uptake of the Alexa Fluor^®^ 647 and the absence of any green color, the obvious reduction in GFP expression. Figure 7c shows HeLa cells 48 h post transfection with AllStars negative control siRNA, the red spots indicate cellular uptake of the tagged siRNA with no noticeable difference in GFP fluorescence relative to the control HeLa cells (Figure 7a). Figure 7c is evidence that the reduction in GFP (seen in Figure 7b and 7d) is due to the sequence specific gene silencing by the siRNA against GFP and not due to cytotoxic or non-specified effects of *N*^4^,*N*^9^-dioleoylspermine **4**.

### 3.5. Cell viability assay

The cell viability assay was carried out in 96-well plates. In order to keep the viability assay conditions as close as possible to the conditions used in the knock down experiments which were carried out in 24-well plates, the amounts of siRNA and cationic lipid, and the number of cells/well were multiplied by the same factor (one-tenth), thus maintaining the N/P ratio and the amount of siRNA per total number of cells constant while downscaling from the 24-well format to the 96-well format. Thus, the 24-well format used 15 pmol siRNA/well, 6 μg cationic lipid/well, and 65,000 cells/well, and the 96-well format used 1.5 pmol siRNA/well, 0.6 μg cationic lipid/well, and 6,500 cells/well.

**Figure 8.**
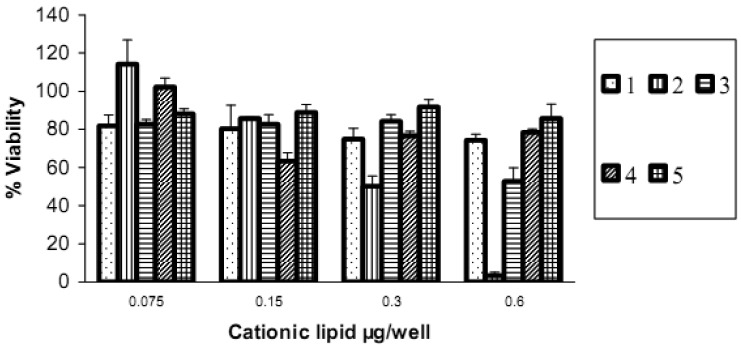

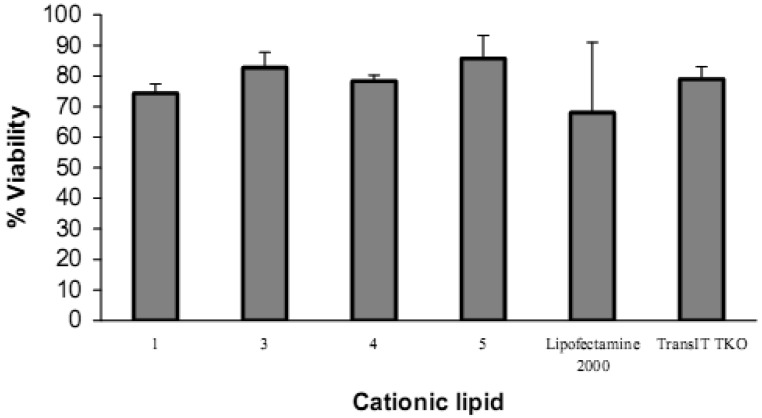
**Upper**: Cell viability alamarBlue assay of fatty acid amides of spermine at different cationic lipid/siRNA ratios. siRNA concentration is kept constant at 1.5 pmol/well (n = 6). **Lower**: Comparison of cell viability of HeLa cells after transfection with fatty acid amides of spermine at their optimal cationic lipid/siRNA ratios and compared with the commercially available transfection agents Lipofectamine 2000 and TransIT TKO Values are presented as mean ± SD (n = 6) using erucoyl (0.6 μg/well), linoleoyl (0.15 μg/well), oleoyl (0.6 μg/well), and stearoyl (0.6 μg/well).

Cell viability evaluation after transfection was carried out with the alamarBlue assay. It can be seen from the upper panel in Figure 8 that increasing the amount of lipid vector (from 0.075 μg to 0.6 μg) was accompanied by a decrease in cell viability. Short chain **2** (lauroyl, 12:0) was much more toxic than the other four spermine conjugates, at 6 μg of **2** there was only 3% cell viability. The cell viability with dilauroyl **2** is lower than that of *N*^4^,*N*^9^-dioleoyl **4** or distearoyl **5** spermine used to transfect HtTA cell line with a scrambled (negative control) fluorescein-labeled siRNA [17]. Indeed, we showed, using the MTT cell viability assay, that the fatty acid conjugates of spermine with shorter chain length (C12-C16) resulted in lower cell viability than the C18 fatty acid spermine conjugates [22]. The lower panel in Figure 8 shows that, with the exception of **2**, our fatty acid spermine amides were at least as well tolerated as the commercially available reagents Lipofectamine 2000 and TransIT TKO in HeLa cells.

## 4. Conclusions

Saturated short chain (C12:0) **2** showed concentration dependant toxicity when compared with the longer chain (C18-C22) spermine fatty acid amides. Transfection with lipoplexes of siRNA and *N*^4^,*N*^9^-difatty acid amides of spermine resulted in successful transfection and gene silencing of GFP in HeLa cells. Varying the chain length or oxidation state of the fatty acids affected both the siRNA delivery and gene silencing efficiency with non-saturated fatty acids (C18:1), (C18:2), and (C22:1) being more efficient compared to saturated fatty acids (C12:0) and (C18:0). There was no evidence for a direct relationship between the amount of siRNA delivered and the extent of gene silencing. *N*^4^,*N*^9^-dilinoleoyl spermine **3** and *N*^4^,*N*^9^-dioleoyl spermine **4** were at least as efficient and as well tolerated (high cell viability) as the commercially available market leaders Lipofectamine 2000 and TransIT TKO. These optimized SAR studies demonstrate that *N*^4^,*N*^9^-difatty acid amides of spermine are efficient, non-viral siRNA delivery vectors *in vitro* for HeLa cells.

## Figures and Tables

**Table 1.** *N*^4^,*N*^9^-difatty acid amides of spermine. The fatty acids are described by two numbers separated by a colon, first the chain length and then the number of double bonds. Particle size and **ζ**-potential of fatty acid amides of spermine measured at the cationic lipid/siRNA ratios that showed best knock-down.

**Name of compound**	**Fatty acid**	**Description of fatty acid**	**Particle size (nm) ± SD**	**Zeta-potential (mV) ± SD**
*N*^4^,*N*^9^-Dierucoylspermine **1**	Erucic	22:1	192 ± 1	63 ± 4
*N*^4^,*N*^9^-Dilauroylspermine **2**	Lauric	12:0	283 ± 41	25 ± 4
*N*^4^,*N*^9^-Dilinoleoylspermine **3**	Linoleic	18:2	353 ± 8	54 ± 1
*N*^4^,*N*^9^-Dioleoylspermine **4**	Oleic	18:1	247 ± 4	54 ± 1
*N*^4^,*N*^9^-Distearoylspermine **5**	Stearic	18:0	145 ± 24	41 ± 7
